# Poor Prognosis of Diffuse Large B-Cell Lymphoma with Hepatitis C Infection

**DOI:** 10.3390/jpm11090844

**Published:** 2021-08-27

**Authors:** Yu-Fen Tsai, Yi-Chang Liu, Ching-I Yang, Tzer-Ming Chuang, Ya-Lun Ke, Tsung-Jang Yeh, Yuh-Ching Gau, Jeng-Shiun Du, Hui-Ching Wang, Shih-Feng Cho, Chin-Mu Hsu, Pey-Fang Wu, Ching-I Huang, Chung-Feng Huang, Ming-Lung Yu, Chia-Yen Dai, Hui-Hua Hsiao

**Affiliations:** 1 Department of Hematology & Oncology, E-Da Cancer Hospital, Kaohsiung 824, Taiwan; 970384kmuh@gmail.com; 2 School of Chinese Medicine for Post Baccalaureate, College of Medicine, I-Shou University, Kaohsiung 824, Taiwan; 3 Division of Hematology and Oncology, Department of Internal Medicine, Kaohsiung Medical University Hospital, Kaohsiung 807, Taiwan; ycliu@cc.kmu.edu.tw (Y.-C.L.); febeey0118@gmail.com (C.-I.Y.); benjer6@gmail.com (T.-M.C.); a9601082@gmail.com (Y.-L.K.); aw7719@gmail.com (T.-J.Y.); cheesecaketwin@gmail.com (Y.-C.G.); ashiun@gmail.com (J.-S.D.); joellewang66@gmail.com (H.-C.W.); sifong96@gmail.com (S.-F.C.); e12013@gmail.com (C.-M.H.); 4 Faculty of Medicine, Kaohsiung Medical University, Kaohsiung 807, Taiwan; Tom65222@gmail.com (C.-I.H.); huangcf@kmu.edu.tw (C.-F.H.); fish6069@gmail.com (M.-L.Y.); daichiayen@gmail.com (C.-Y.D.); 5 Specialist Nurse and Surgical Nurse Practitioner Office, Kaohsiung Medical University Hospital, Kaohsiung Medical University, Kaohsiung 807, Taiwan; 6 Division of Hepatobiliary Ward, Department of Internal Medicine, Kaohsiung Medical University Hospital, Kaohsiung 807, Taiwan; Fang820018@gmail.com; 7 Center for Liquid Biopsy, Kaohsiung Medical University, Kaohsiung 807, Taiwan; 8 Cancer Center, Kaohsiung Medical University Hospital, Kaohsiung Medical University, Kaohsiung 807, Taiwan

**Keywords:** hepatitis C virus, diffuse large B-cell lymphoma, survival, fibrosis, performance status, liver toxicity

## Abstract

Background: Hepatitis C virus (HCV) in diffuse large B-cell lymphoma (DLBCL) is associated with a higher prevalence and distinctive clinical characteristics and outcomes. Methods: A retrospective analysis of adult DLBCL patients from 2011 to 2015 was studied. Results: A total of 206 adult DLBCL were enrolled with 22 (10.7%) HCV-positive patients. Compared to HCV-negative patients, the HCV-positive group had a poor performance status (*p* = 0.011), lower platelet count (*p* = 0.029), and higher spleen and liver involvement incidences (liver involvement, *p* = 0.027, spleen involvement, *p* = 0.026), and they received fewer cycles of chemotherapy significantly due to morbidity and mortality (*p* = 0.048). Overall survival was shorter in HCV-positive DLBCL (25.3 months in HCV-positive vs. not reached (NR), *p* = 0.049). With multivariate analysis, poor performance status (*p* < 0.001), advanced stage (*p* < 0.001), less chemotherapy cycles (*p* < 0.001), and the presence of liver toxicity (*p* = 0.001) contributed to poor OS in DLBCL. Among HCV-positive DLBCL, the severity of liver fibrosis was the main risk factor related to death. Conclusion: Inferior survival of HCV-positive DLBCL was observed and associated with poor performance status, higher numbers of complications, and intolerance of treatment, leading to fewer therapy. Therefore, anti-HCV therapy, such as direct-acting antiviral agents, might benefit these patients in the future.

## 1. Introduction

Hepatitis C virus (HCV) infection is one of the major causes of chronic liver disease around the world with a prevalence rate of around 3% [1]. In addition to hepatic function, various extrahepatic manifestations, including vasculitis, glomerulonephrisis, and a wide range of lymphoproliferative disorders, have been associated with HCV infections, resulting in further morbidities and mortalities [2,3]. HCV infection are also associated with B-cell non-Hodgkin lymphoma (NHL) with distinct clinical presentation and outcome, especially in diffuse large B-cell lymphoma (DLBCL) and marginal zone lymphoma (MZL) in recent epidemiological studies [4,5,6,7,8,9]. Due to the fact that direct anti-HCV therapy induces hematologic response in indolent NHL patients with HCV infection, HCV infection has been linked to NHL, and front-line therapy of asymptomatic indolent NHLs is recommended [10,11]. DLBCL, the most common NHL, is an aggressive type of NHL with specific genetic features and clinical presentations [12]. Although HCV-positive DLBCLs display specific clinical features and outcomes, the optimal treatment and timing of anti-HCV therapy in aggressive lymphoma is still uncertain [1,13,14,15,16]. Previous reports showed that HCV-positive patients with DLBCL exhibited worse overall survival (OS), and the incidence of severe hepatic toxicity in HCV-positive patients was significantly higher than that of HCV-negative patients [13,17]. However, all these studies only included small numbers of HCV-positive patients, and not all studies regarding HCV infection and DLBCL showed consistent results. 

In addition, the seroprevalence of anti-HCV antigen in Taiwan is around 1 to 5.4% in the general population [18,19,20], and a retrospective study that investigated the association of HCV and lymphoma in southern Taiwan by Chuang et al. displayed that the incidence of HCV infection among lymphoma patients was significantly higher than that in healthy controls (11.0% vs. 1.8%, *p* < 0.001) [21]. The differences of HCV infection in NHL patients reflect geographic differences in HCV epidemiology [1,22,23]. The purpose of this study is to analyze clinicopathological characteristics, tolerance to chemotherapy, and clinical outcomes of DLBCL patients between HCV-positive and HCV-negative groups in order to determine the impact of HCV infection in this population.

## 2. Materials and Methods

### 2.1. Study Population

In this retrospective observational study, newly diagnosed DLBCL patients who were managed at Kaohsiung Medical University Hospital between January 2011 and December 2015 were enrolled. Clinical characteristics, including patients’ demographics, baseline laboratory and biochemical data, hepatitis virus serology including hepatitis B virus (HBV) and HCV, and FIB (fibrosis)-4 index were collected and analyzed from medical records. Lymphoma was treated according to the treatment guideline in the hospital. The study was approved by the Institutional Review Board of Kaohsiung Medical University Hospital (KMUHIRB-E(I)-20210119).

### 2.2. Risk Groups and Outcomes 

Patients were categorized as HCV-positive or HCV-negative groups based on the presence or absence of anti-HCV antibody. Since the interferon-free direct-acting antiviral agent (DAA) regimens were added to the reimbursement list in Taiwan for HCV treatment in 2017, patients were only followed until 2017 to eliminate the confounding factor of DAA treatment. All HCV-positive patients in this study did not receive interferon-free DAA prior to or during chemotherapy. All DLBCL patients with HBsAg-positive received antiviral agents for HBV prophylaxis. Performance status was evaluated by Eastern Cooperative Oncology Group (ECOG) scale [24]. The FIB-4 index was used to assess the severity of liver fibrosis as it is considered to be a useful fibrosis scoring system in HCV. A cutoff of >3.25 had a positive predictive value of 65% and a specificity of 97% to predict advanced fibrosis [25,26]. In Taiwan, liver cirrhosis was diagnosed by the image, either sonography or computed tomography or by FIB-4 index ≥ 6.5. The international prognostic index (IPI) score divided patients into four groups: low, low-intermediate, high-intermediate, and high for risk stratification [27], and the revised international prognostic index (R-IPI) classified patients into three groups: very good, good, and poor [28], which was calculated and used for analysis. The treatment response was evaluated by computed tomography (CT) or positron emission tomography–computed tomography (PET-CT) every 3–6 months according to response criteria [29]. The first time of treatment response evaluation was at the time after 3–4 courses of chemoimmunotherapy. The liver toxicity was calculated by the common terminology criteria for adverse events (CTACE), version 4. Progression-free survival (PFS) was defined as the length of time from the date of diagnosis to the date of progression or death. Overall survival (OS) was defined as the length of time from the date of diagnosis to the last date of follow-up or death. Liver function was monitored during every course of treatment during chemotherapy and around every 3 months after chemotherapy. 

### 2.3. Statistical Analysis

Patient characteristics between HCV-positive and HCV-negative groups were analyzed using descriptive statistics and are presented as frequencies, percentages, and means with standard deviations. Student’s t-test or nonparametric statistics were utilized to test for statistically significant differences in continuous variables, whereas the chi-square or Fisher’s exact test was used for categorical variables. OS and PFS was estimated using the Kaplan–Meier method. Variables with a *p*-value of less than 0.05 in the univariate analysis of overall survival were subsequently subjected to multivariate analysis using a Cox regression model. A two-tailed *p*-value < 0.05 was considered statistically significant.

## 3. Results

### 3.1. Baseline Characteristics

A total of 206 DLBCL patients were enrolled in the study. Among them, twenty-two (10.7%) were HCV-positive. The characteristics at the time of diagnosis are listed in Table 1. Compared to HCV-negative patients, HCV-positive patients seemed to have a significantly lower platelet count (186.7 ± 68.8 × 103/μL vs. 236.2 ± 102.9 × 103/μL, *p* = 0.029) and a trend of older age (mean age 67.23 ± 17.13 vs. 61.24 ± 15.27, *p* = 0.088). HCV-negative patients had better performance status compared to HCV-positive patients (*p* = 0.011). Higher spleen and liver involvement rates were observed in HCV-positive patients compared to HCV-negative (liver involvement: 18.2% in HCV-positive vs. 4.3% in HCV-negative, *p* = 0.027, spleen involvement: 31.8% in HCV-positive vs. 13.6% in HCV-negative, *p* = 0.026). More HCV-positive patients had elevated aminotransferase compared to HCV-negative population, however, not to a statistical significant degree (31.8% vs. 16.3%, *p* = 0.073). There were no significant differences on sex, B symptoms, bone marrow involvement, IPI score, R-IPI score, HBsAg status, white blood cell (WBC) count, hemoglobin, albumin, lactate dehydrogenase (LDH) levels, alanine aminotransferase (GPT) levels, Beta-2 macroglobulin levels, and the percentage of receiving treatment between two groups.

### 3.2. Response to Therapy and Outcomes

Patients were managed by guidelines according to clinical status. Most (92.9%) of the HCV-negative patients and 86.4% of the HCV-positive patients received treatment respectively without statistical difference (*p* = 0.388) with most of them (74.3% in HCV-negative, 68.4% in HCV-positive) receiving an R-CHOP (rituximab, cyclophosphamide, daunorubicin, oncovin, and prednisolone) regimen. The details of the chemotherapy regimen were presented in Table 2, and there was no significant difference in treatment regimen between the HCV-positive and HCV-negative population. The HCV-positive population received fewer cycles of chemotherapy compared with HCV-negative patients (mean cycles of chemotherapy: 4.8 ± 3.5 in HCV-positive vs. 6.3 ± 3.4 in HCV-negative, *p* = 0.048) (Table 2). A lower evaluation of treatment response during the follow-up period was found in HCV-positive patients compared to the HCV-negative patient group (31.8% in HCV positive vs. 14.7% in HCV-negative, *p* = 0.041). Among these HCV-positive patients who were unable to be evaluated, four of seven patients received limited treatment with three of them receiving only one cycle of chemotherapy due to early mortality from sepsis and/or other morbidities. Three of seven patients had supportive management due to old age (*n* = 2, over 75 years old) and severe liver cirrhosis (*n* = 1).

The progression and recurrent events were similar in HCV-positive and HCV-negative groups in evaluable patients (27.3% in HCV-positive vs. 28.3% in HCV-negative, *p* = 0.922). Higher incidence of liver toxicity (86.4% in HCV-positive vs. 45.1% in HCV-negative, *p* < 0.001) and severe liver toxicity (≥grade 3 liver toxicity, 36.4% in HCV-positive vs. 9.8% in HCV-negative, *p* < 0.001) were observed in HCV-positive patients than HCV-negative patients. The incidence of liver toxicity was not associated with baseline GPT levels in HCV-positive patients (20.67 IU/mL, without liver toxicity vs. 37.16 IU/mL, with liver toxicity, *p* = 0.366). More patients in the HCV-positive group delayed or discontinued their treatment due to toxicities than in the HCV-negative group (40.9% vs. 12.5%, *p* = 0.001) 

**Figure 1:**
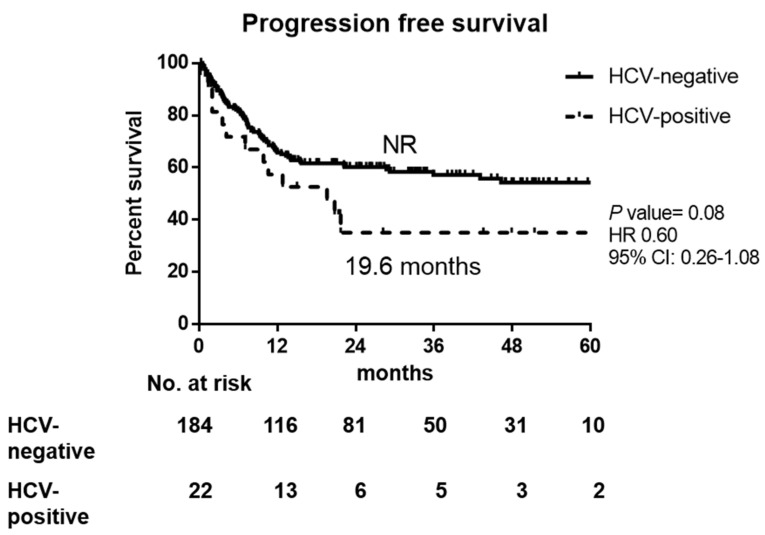
Progression-free survival of HCV-positive and HCV-negative DLBCL patients. NR: not reached, HR: hazard ratio, CI: confidence interval.

**Figure 2:**
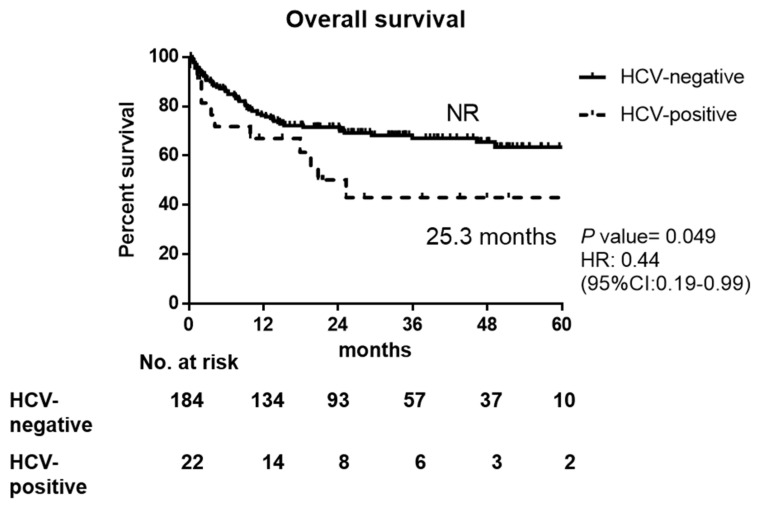
Overall survival of HCV-positive and HCV-negative DLBCL patients. NR: not reached, HR: hazard ration, CI: confidence interval.

There was a trend of worse PFS in HCV-infected patients than non-infected, but no statistical significance (19.6 months in HCV-positive vs. NR in HCV-negative, *p* = 0.080), as shown in Figure 1. The 1-year, 2-year, and 3-year OS rates in HCV-positive and HCV-negative DLBCL patients were 67.0% vs. 76.4%, 50.2% vs. 71.6%, and 43.1% vs. 67.0%, respectively. The OS was worse in HCV-positive DLBCL patients (25.3 months in HCV-positive vs. not reached (NR) in HCV-negative, *p* = 0.049 by Kaplan–Meier method, Figure 2), which might result from early mortality, higher liver toxicity, and higher incidence of treatment delay/discontinuation with fewer treatment courses.

### 3.3. Prognostic Factors for Overall Survival and Progression Free Survival in DLBCL Patients

Based on univariate analysis via Cox regression model, we found that older age (HR: 1.03, 95% CI: 1.01–1.04, *p* = 0.003), poor performance status (ECOG ≥ 2, HR: 6.22, 95% CI: 3.66–10.56, *p* < 0.001), advanced stage (HR 4.36, 95% CI: 2.23–8.55, *p* <0.001), less chemotherapy cycles (HR: 0.77, 95% CI: 0.70–0.84, *p* < 0.001), and the presence of liver toxicity (HR: 2.46, 95% CI: 1.47–4.12, *p* = 0.001) contributed to poor OS. In multivariate analysis, four key factors: advanced stage, poor performance status, liver toxicity presentation, and less chemotherapy cycles were independent poor prognostic factors for overall survival in DLBCL patients (Table 3). Regarding the progression-free survival, we found that after multivariate analysis, poor performance status (ECOG ≥ 2, HR: 2.82, 95% CI: 1.56–5.09, *p* < 0.001), advanced stage (HR: 3.18, 95% CI: 1.81–5.58, *p* < 0.001), the presence of liver toxicity (HR: 2.67, 95% CI: 1.69–4.22, *p* < 0.001), and less chemotherapy cycles (HR 0.90, 95% CI: 0.83–0.98, *p* = 0.015), contributed to poor PFS in DLBCL patients (Table 4).

### 3.4. Details of HCV-Positive DLBCL Patients during Follow-Up Period

The details of HCV-positive DLBCL patients are listed in Table S1. Half of HCV-positive (11 of 22) DLBCL patients died during the follow-up period. Seven died of sepsis, two died due to the progression of hepatocellular carcinoma, one died of gastrointestinal bleeding related to liver cirrhosis, and only one patient died of lymphoma progression. Based on subgroup analyses of the death events in the HCV-positive population, no difference of initial performance status, stage, IPI score, platelet count, coagulation profile (prothrombin time, PT), albumin, GPT, and LDH levels was found between the dead and alive groups. However, less chemotherapy cycles, higher percentage of severe liver toxicity, and treatment delay/discontinuation were observed in the death population, but not to a statistically significant degree. We found that only liver cirrhosis status contributed to more death events in HCV-positive patients. In HCV-positive patients, all were alive of those without liver cirrhosis (*p* = 0.035) or FIB-4 index less than 1.45 points (*p* = 0.032) (Table 5). In addition, only one of 22 HCV-positive patients started peg-interferon and ribavirin treatment for HCV at 4 months after the end of chemotherapy, and HCV viral load was 2758.2 IU/mL at that time. The HCV viral load became undetectable after peg-interferon and ribavirin status to now.

## 4. Discussion

In our study, the prevalence of HCV infection was 10.7% (22/206 patients) in DLBCL patients. It was higher than the prevalence rate of the general population in Taiwan at 4.4% (6904/157,720) of anti-HCV positive [30]. There have been some hypothetical models to describe the possible pathologic role of HCV infection in aggressive B-cell lymphoma. The direct transformation mechanism is one kind of hypothesis accounting for HCV-associated lymphomagenesis. In vitro, the infection of B-cell lines with HCV leads to somatic mutations of several oncogenes and tumor-suppressor genes such as p53, beta-catenin, and Bcl6 [31]. On the contrary, some studies support the role of HCV as an indirect transformation agent by chronically stimulating B-cell immunologic response and finally leading to lymphoma. Polyclonal or monoclonal B-cell proliferation can be detected in the blood or bone marrow of HCV patients and is associated with mixed cryoglobulinemia type II. The risk of lymphoma in patients with HCV-associated cryoglobulinemia is estimated to be 35 times higher than that in the general population [32]. All these theories are proposed to explain the high prevalence of HCV infection in lymphoma patients.

In previous retrospective studies, HCV-infected DLBCL patients shared distinctive clinical features. A higher percentage of HCV-positive DLBCL cases were associated with old age (> or =60) than HCV-negative DLBCL cases at diagnosis [14,33]. HCV-positive patients had more frequent extra-nodal involvement [13,33] and more frequent elevated lactate dehydrogenase (LDH) levels than other patients [13]. In our studies, we found that HCV-positive DLBCL patients had poorer performance status, lower platelet count, and higher probability of liver and spleen involvement than HCV-negative patients. In terms of OS and PFS, the influence of HCV infection on survival in DLBCL patients remains controversial. Studies conducted by Ennishi et al. reported a similar outcome in HCV-positive DLBCL patients compared to HCV-negative (3-year OS 75% in HCV-positive vs. 84% in HCV-negative, *p* = 0.07) [17]. On the contrary, two other studies presented an inferior outcome in HCV-positive patients [16,34]. Chen et al. found that three independent factors predicted a dismal OS: lower albumin level (<3 g/dL vs. ≥3 g/dL; HR = 13.21, 95% CI = 2.69–64.98, *p* = 0.001), presence of HCV infection (HCV-positive vs. HCV-negative; HR = 9.75, 95% CI = 1.97–48.34, *p* = 0.005), and poor IPI risk (high-intermediate or high vs. low-intermediate or low; HR = 5.56, 95% CI = 1.17–26.55, *p* = 0.031) [34]. The Fondazione Italiana Linfomi has conducted a multicenter retrospective study to explore a new prognostic system for HCV-associated DLBCL. Therefore, the HCV Prognostic Score, based on performance status, albumin level, and HCV-RNA viral load, was therefore introduced as a useful tool to predict the outcome of HCV-associated DLBCL [16]. 

In our study, we found that an inferior overall survival was observed in HCV-positive patients by the Kaplan–Meier method. However, after multivariate analysis, HCV infection status was not the key risk factor for overall survival. Instead, advanced stage, poor performance status, liver toxicity presentation, and less chemotherapy cycles were the main risk factors that predicted overall survival and progression-free survival. A higher percentage of HCV-positive patients’ treatment response was unable to be evaluated during the follow-up period (31.8% in HCV-positive vs. 14.7% in HCV-negative, *p* = 0.041). Nearly half (42.9%, three of seven) of them did not receive any treatment due to elderly age with poor performance status and severe liver cirrhosis; the rest of the patients received treatment of no more than two cycles with no possible follow-up image due to sepsis-related death. This highlighted the ability of HCV co-morbidity to hamper treatment completeness. The number of chemotherapy cycles was a major predictive factor for OS and HCV-positive patients; fewer chemotherapy cycles resulted in poor survival in the HCV-positive group. The phenomenon was also observed by Dlouhy et al., who presented higher number of complications and early deaths in HCV-positive DLBCL patients [33].

Regarding the HCV-positive DLBCL population, we found that liver cirrhosis was the main factor related to death events. Unlike the whole DLBCL population, age, stage, and performance status carried no vital role in predicting survival in HCV-positive DLBCL patients. Although fewer chemotherapy cycles, higher percentage of severe liver toxicity, and treatment delay/discontinuation were observed among those patients that died, the difference was not statistically significant compared to those who remained alive. While these findings highlighted the influence of the severity of liver cirrhosis for HCV-positive DLBCL patients, the small sample size might limit the statistical findings. This result highlighted that the severity of cirrhosis was the major factor to determine survival in HCV-positive DLBCL patients and also contributed to high complications and early deaths in HCV-positive DLBCL patients.

We found higher liver toxicity and higher ≥ grade 3 liver toxicity in HCV-positive population, and the incidence of liver toxicity was not associated with initial GPT levels. One patient (4.5%, one of 22 HCV-positive) received peg-interferon and ribavirin treatment for high HCV RNA levels after chemoimmunotherapy. Since we did not have the comprehensive data of HCV viral load, it was difficult to be conclude as a HCV reactivation in our study. The increased risk of liver toxicity in the HCV-positive population was consistent with other studies. In a Japanese multicenter retrospective study of 553 DLBCL patients, HCV RNA levels significantly increased in the HCV-positive patients during immunochemotherapy, and more grade 3–4 hepatic toxicity (27%) was observed in patients with HCV-positive patients than HCV-negative patients (3%) [17]. In another Italian retrospective study of 156 HCV-positive DLBCL patients, they found that 85% of patients completed their therapeutic program without any interruption or dosage reduction and five patients (4%) had to discontinue chemotherapy due to severe hepatic function impairment (toxicity grade 3–4) [35]. These studies revealed that without initial liver dysfunction, HCV-positive patients experience a similar outcome compared to HCV-negative patients when treated with chemotherapy or immunotherapy [36]. Therefore, careful monitoring of hepatic function and viral load is suggested during the therapy, especially for those with initial liver dysfunction patients.

Some studies showed that DAA treatment could reverse the liver inflammation, fibrosis, and decrease the complications of compensated liver cirrhosis in chronic hepatitis C [37,38,39]. From the observation report of Occhipinti et al., seven DLBCL patients with HCV infection received different DAA regimens concurrently with immunochemotherapy [40]. All patients completed their scheduled treatment with no liver toxicity occurrence. Thus, the concomitant administration of DAA and immunochemotherapy for HCV-positive DLBCL seems safe and may prevent immunochemotherapy-induced liver toxicity. Further prospective studies are warranted to identify if concurrent DAA therapy with chemoimmunotherapy in HCV-positive DLBCL patients can lead to survival benefit, especially for those with advanced fibrosis.

There were some limitations in this study. First, our study was a retrospective study with limited numbers of HCV patients, which limits the scope of our conclusion. Secondly, HCV viral load was not routinely checked during 2011–2015. Therefore, we did not have the comprehensive data of HCV viral load in all HCV-positive patients. Thus, we only used the anti-HCV positivity as criteria for being HCV-positive. Thirdly, some patients had a shortened course of therapy due to mortalities, which hinders proper response evaluations, especially in HCV-positive patients. However, this study presents real-world data of clinical characteristics and outcomes of HCV-positive DLBCL patients before the introduction of DAA treatment and provided the association of HCV and DLBCL.

## 5. Conclusions

In our study, DLBCL patients have a higher prevalence (10.7%) of HCV infection than the general population in our area. Clinically, HCV-positive DLBCL patients had poor performance status, lower platelet count, higher probability of spleen and liver involvement, and received less chemotherapy cycles due to morbidity and mortality. With multivariate analysis, stage, performance status, liver toxicity presentation, and chemotherapy cycles were found to be the major factors for the OS and PFS in DLBCL patients. Inferior survival of HCV-positive DLBCL was contributed by poor performance status, higher numbers of complications, and intolerance of treatment, leading to fewer therapy. Severity of liver fibrosis was the main factor related to death in the HCV-positive population. As such, anti-HCV therapy such as direct-acting antiviral agents might benefit such patients in the future to improve outcomes.

## Figures and Tables

**Table 1:** Baseline characteristics of HCV-negative and HCV-positive DLBCL patients.

Variables	HCV-Negative DLBCL	HCV-Positive DLBCL	
	N = 184	N = 22	*p* Value
Age, (Mean ± SD)	61.24 ± 15.27	67.23 ± 17.13	0.088
Gender, N(%)			0.618
Male	94(51.1%)	10(45.5%)	
Female	90(48.9%)	12(54.5%)	
ECOG, N(%)			0.011 *
0	79(42.9%)	6(27.3%)	
1	78(42.4%)	12(54.5%)	
2	13(7.1%)	1(4.5%)	
3	11(6.0%)	0(0%)	
4	3(1.6%)	3(13.6%)	
Ann Arbor stage, N(%)			0.957
I-II	68(37.0%)	8(36.4%)	
III-IV	116(63.0%)	14(63.6%)	
B symptoms, N(%)	74(40.2%)	13(59.1%)	0.090
Liver involvement, N(%)	8(4.3%)	4 4(18.2%)	0.027 *
Spleen involvement, N(%)	25(13.6%)	7(31.8%)	0.026 *
Bone marrow involvement, N(%)	38(20.7%)	4(18.2%)	1.000
R-IPI score, N(%)			0.202
Very good	15(8.2%)	0(0%)	
Good	90(48.9%)	9(40.9%)	
Poor	79(42.9%)	13(59.1%)	
IPI score, N(%)			0.448
Low	57(31.0%)	6(27.3%)	
Low-intermediate	48(26.1%)	3(13.6%)	
High-intermediate	41(22.3%)	6(27.3%)	
High	38(20.7%)	7(31.8%)	
HBsAg positive, N(%)	38(20.7%)	2(9.1%)	0.261
Abnormal liver funtion at baseline, N(%)	30(16.3%)	7(31.8%)	0.073
WBC (Mean ± SD)	7554.6 ± 3474.0	7480.0 ± 2823.8	0.923
Hgb (Mean ± SD)	11.8 ± 2.3	11.6 ± 1.7	0.687
Platelet (×10^3^, Mean ± SD)	236.2 ± 102.9	186.7 ± 68.8	0.029 *
Albumin (Mean ± SD)	3.6 ± 0.6	3.6 ± 0.6	0.969
LDH (Mean ± SD)	366.9 ± 453.2	430.9 ± 450.3	0.532
GOT (Mean ± SD)	48.2 ± 134.5	58.1 ± 77.5	0.734
GPT (Mean ± SD)	43.7 ± 135.0	34.9 ± 28.6	0.763
Beta-2 microglobulin (Mean ± SD)	303.5 ± 273.2	332.9 ± 116.1	0.627

*: *p* < 0.05, ECOG: Eastern Cooperative Oncology Group, R-IPI: revised international prognostic index, IPI: international prognostic index, SD: standard deviation, WBC: white blood cell, Hgb: hemoglobin, LDH: lactate dehydrogenase, GPT: alanine aminotransferase.

**Table 2:** Treatment detail, response, and liver toxicity in HCV-negative and HCV-positive DLBCL patients.

Variables	HCV-Negative DLBCL	HCV-Positive DLBCL	
	N = 184	N = 22	*p* Value
Receiving treatment (Yes), N(%)	171(92.9%)	19(86.4%)	0.388
First-line treatment regimen in receiving treatment population			0.354
RCHOP	127(74.3%)	13(68.4%)	
RCOP	30(17.5%)	6(31.6%)	
Other regimen (REPOCH, RMTX)	11(6.4%)	0(0.0%)	
R with prednisolone	3(1.8%)	0(0.0%)	
Chemotherapy cycles (Mean ± SD)	6.3 ± 3.4	4.8 ± 3.5	0.048 *
Treatment response, N(%)			
Complete response/Partial response	105(57.1%)	9(40.9%)	0.150
Progression or recurrent	52(28.3%)	6(27.3%)	0.922
Unable to evaluate response	27(14.7%)	7(31.8%)	0.041 *
Liver toxicity	83(45.1%)	19(86.4%)	<0.001 *
≥Grade 3 liver toxicity	18(9.8%)	8(36.4%)	<0.001 *
Treatment delay or discontinue	23(12.5%)	9(40.9%)	0.001 *

* *p* < 0.05, R-CHOP: rituximab, cyclophosphamide, daunorubicin, oncovin and prednisolone; R-COP: rituximab, cyclophosphamide, oncovin and prednisolone; R-EPOCH: rituximab, etoposide, cyclophosphamide, daunorubicin, oncovin and prednisolone; R: rituximab.

**Table 3:** Univariate and multivariate analysis of risk factors for overall survival.

	Univariate Analysis		Multivariate Analysis ^a^	
Variables	HR (95%CI)	*p*-Value	HR (95%CI)	*p*-Value
Age	1.03(1.01–1.04)	0.004 *	1.00(0.98–1.02)	0.733
Gender		0.479		
Male	1.0			
Female	0.84(0.52–1.36)			
Hepatitis C infection		0.054		0.574
Negative	1.0		1.00	
Positive	1.89 (0.99–3.60)		0.82(0.41–1.64)	
Hepatitis B infection		0.069		0.202
Negative	1		1.00	
Positive	0.50(0.24–1.06)		0.61(0.28–1.31)	
ECOG		<0.001 *		0.003 *
<2	1.00		1.00	
≥2	6.22(3.66–10.56)		2.64(1.39–4.99)	
Stage		<0.001 *		<0.001 *
I/II	1.0		1.00	
III/IV	4.36(2.23–8.55)		4.28(2.12–8.63)	
Chemotherapy cycles	0.77(0.70–0.84)	<0.001 *	0.78(0.70–0.86)	<0.001 *
Liver toxicity		0.001 *		<0.001 *
Yes	2.46(1.47–4.12)		2.73(1.62–4.60)	
No	1.00		1.00	

*: *p* < 0.05, HR: hazard ratio, CI: confidence interval, ECOG: Eastern Cooperative Oncology Group. ^a^: Multivariate analyses: variables with *p* < 0.1 in univariate analyses were included in multivariate analyses.

**Table 4:** Univariate and multivariate analysis of risk factors for progression-free survival.

	Univariate Analysis		Multivariate Analysis ^a^	
Variables	HR (95%CI)	*p*-Value	HR (95%CI)	*p*-Value
Age	1.02(1.00–1.03)	0.033 *	1.00(0.98–1.01)	0.838
Gender		0.184		
Male	1.0			
Female	0.75(0.49–1.15)			
Hepatitis C infection		0.084		0.827
Negative	1.0		1.00	
Positive	1.68 (0.93–3.03)		0.93(0.50–1.74)	
Hepatitis B infection		0.025 *		0.041 *
Negative	1		1.00	
Positive	0.47(0.24–0.91)		0.49(0.25–0.97)	
ECOG		<0.001 *		0.001 *
<2	1.00		1.00	
≥2	4.60(2.81–7.53)		2.82(1.56–5.09)	
Stage		<0.001 *		<0.001 *
I/II	1.0		1.00	
III/IV	3.59(2.09–6.18)		3.18(1.81–5.58)	
Chemotherapy cycles	0.89(0.82–0.96)	0.003 *	0.90(0.83–0.98)	0.015 *
Liver toxicity		0.001 *		<0.001*
Yes	2.56(1.63–4.02)		2.67(1.69–4.22)	
No	1.00		1.00	

*: *p* < 0.05, HR: hazard ratio, CI: confidence interval, ECOG: Eastern Cooperative Oncology Group ^a^: Multivariate analyses: variables with *p* < 0.1 in univariate analyses were included in multivariate analyses.

**Table 5:** Different variables in the dead and alive groups of HCV-positive DLBCL patients.

Variables	HCV-Positive DLBCL		
	Dead (*n* = 11)	Alive (*n* = 11)	*p*-Value
Age, (Mean ± SD)	69.00 ± 14.11	65.45 ± 20.25	0.639
Sex			0.198
Male	3(27.3%)	7(63.6%)	
Female	8(72.7%)	4(36.4%)	
ECOG			1.000
<2	9(81.8%)	9(81.8%)	
≥2	2(18.2%)	2(18.2%)	
Ann Arbor stage			1.000
I–II	4(36.4%)	4(36.4%)	
III–IV	7(63.6%)	7(63.6%)	
IPI score			0.924
Low	3(27.3%)	3(27.3%)	
Low-intermediate	1(9.1%)	2(18.2%)	
High-intermediate	3(27.3%)	3(27.3%)	
High	4(36.4%)	3(27.3%)	
GPT at diagnosis (Mean ± SD)	42.82 ± 36.68	27.00 ± 15.29	0.209
PLT at diagnosis (×10^3^, Mean ± SD)	184.45 ± 72.12	188.91 ± 68.80	0.884
Albumin at diagnosis (Mean ± SD)	3.53 ± 0.74	3.63 ± 0.58	0.737
PT(INR) at diagnosis (Mean ± SD)	1.05 ± 0.78	1.04 ± 0.53	0.707
LDH at diagnosis (Mean ± SD)	531.45 ± 615.90	330.45 ± 155.97	0.307
Abnormal liver funtion at baseline, N(%)	5(45.5%)	2(18.2%)	0.361
Liver cirrhosis			0.035 *
No	6(54.5%)	11(100%)	
Yes	5(45.5%)	0(0%)	
FIB-4 index			0.032 *
<1.45	0(0%)	4(36.4%)	
1.45–3.25	4(36.4%)	5(45.5%)	
>3.25	7(63.6%)	2(18.2%)	
Receiving treatment			1.000
Yes	9(81.8%)	10(90.9%)	
No	2(18.2%)	1(9.1%)	
Chemotherapy cycles	3.91 ± 4.04	5.64 ± 2.77	0.255
≥Grade 3 Liver toxicity			0.183
Yes	6(54.5%)	2(18.2%)	
No	5(45.5%)	9(81.8%)	
Treatment delay or discontinuation			0.387
Yes	6(54.5%)	3(27.3%)	
No	5(45.5%)	8(72.7%)	

*: *p* < 0.05, SD: standard deviation, ECOG: Eastern Cooperative Oncology Group, IPI: international prognostic index GPT: alanine aminotransferase, PLT: platelet, PT: prothrombin time, INR: international normalized ratio, LDH: lactate dehydrogenase, FIB-4: fibrosis-4.

## Data Availability

The data used in the present study are available from the corresponding author upon reasonable request.

## Supplementary Materials

The following are available online at https://www.mdpi.com/article/10.3390/jpm11090844/s1, Table S1: the details of HCV-positive DLBCL patients.
