# Supplementary material for: Poor Prognosis of Diffuse Large B-Cell Lymphoma with Hepatitis C Infection
Source: J Pers Med. 2021 Aug 27;11(9):844. doi: 10.3390/jpm11090844 (PMC8465128; doi:10.3390/jpm11090844)
Supplement: Supplementary file 1 [file jpm-11-00844-s001.zip › jpm-1313298-supplementary.pdf]

TableS1: The details of HCV-positive DLBCL patients

Case No.	Age	Sex	ECOG	Stage	IPI score	B symptoms	Spleen involvement	Liver involvement	Bone marrow involvement	FIB-4 index	Liver cirrhosis	Lymphoma Treatment	Liver toxicity, (grade)	Status at the end of follow-up	Death reason
1	80	F	1	2	2	No	No	No	No	4.03	No	Yes	Yes(2)	Alive	
2	65	M	0	4	3	Yes	Yes	No	No	3.21	No	Yes	Yes(1)	Alive	
3	75	F	1	4	4	No	No	No	No	2.44	No	Yes	Yes(2)	Alive	
4	71	M	1	3	3	Yes	Yes	No	No	2.80	No	Yes	Yes(2)	Alive	
5	44	M	0	2	1	No	No	No	No	1.06	No	Yes	Yes(1)	Alive	
6	52	M	0	4	3	No	Yes	No	Yes	2.30	No	Yes	Yes(1)	Alive	
7	63	F	0	2	1	Yes	No	No	No	1.35	No	Yes	Yes(3)	Alive	
8	20	M	0	4	2	Yes	Yes	No	Yes	1.43	No	Yes	Yes(3)	Alive	
9	78	M	1	2	1	No	No	No	No	1.98	No	Yes	Yes(1)	Alive	
10	81	M	4	4	5	Yes	Yes	No	No	1.08	No	No	No	Alive	
11	91	F	4	3	5	Yes	No	No	No	5.63	No	Yes	Yes(1)	Alive	
12	78	F	2	4	5	Yes	Yes	Yes	No	3.45	No	Yes	Yes(1)	Dead	Sepsis, infection
13	75	F	1	4	2	Yes	Yes	Yes	No	5.22	Yes, child A	No	No	Dead	Sepsis, infection
14	55	F	1	4	3	Yes	No	Yes	No	7.56	Yes,	No	No	Dead	Sepsis,

											child B				infection
15	83	M	1	3	4	Yes	No	No	No	5.35	Yes, child A	Yes	Yes(3)	Dead	Sepsis, infection
16	83	F	1	1	1	No	No	No	No	5.38	Yes, child A	Yes	Yes(1)	Dead	Sepsis, HCC rupture
17	63	F	1	4	4	No	No	No	Yes	2.77	No	Yes	Yes(4)	Dead	HCC progression
18	67	F	1	2	3	Yes	No	No	No	3.69	Yes, child A	Yes	Yes(3)	Dead	GI bleeding due to liver cirrhosis
19	82	M	1	2	1	No	No	No	No	2.46	No	Yes	Yes(4)	Dead	Sepsis, infection
20	38	M	4	4	4	Yes	No	Yes	No	10.99	No	Yes	Yes(3)	Dead	Lymphoma progression
21	60	F	1	4	3	Yes	No	No	Yes	1.68	No	Yes	Yes(3)	Dead	Sepsis, infection
22	75	F	0	2	1	No	No	No	No	1.63	No	Yes	Yes(1)	Dead	Sepsis, infection

*F: female, M: male, ECOG: Eastern Cooperative Oncology Group, IPI: international prognostic index, FIB-4: fibrosis-4, HCC: hepatocellular carcinoma, GI: gastrointestinal
